# Oceanic Distribution, Behaviour, and a Winter Aggregation Area of Adult Atlantic Sturgeon, *Acipenser oxyrinchus oxyrinchus*, in the Bay of Fundy, Canada

**DOI:** 10.1371/journal.pone.0152470

**Published:** 2016-04-04

**Authors:** Andrew Douglas Taylor, Kyoko Ohashi, Jinyu Sheng, Matthew Kenneth Litvak

**Affiliations:** 1 Department of Biology, Mount Allison University, Sackville, New Brunswick, Canada; 2 Department of Oceanography, Dalhousie University, Halifax, Nova Scotia, Canada; Technical University of Denmark, DENMARK

## Abstract

Seasonal distribution of adult Atlantic sturgeon was examined using pop-up satellite archival tags (PSATs) and ultrasonic transmitters deployed in the Saint John River, New Brunswick, Canada. Seven MK10 PSATs programmed for release in June 2012 and seven MiniPAT PSATs programmed for release in February and April 2013 were deployed in August 2011 and 2012, respectively. Eleven of 14 PSATs surfaced and transmitted depth and temperature data archived for the duration of their deployment (121–302 days). Among these eleven PSATs, five were recovered and 15-sec archival data was downloaded. Following exit from the Saint John River in the fall, tagged fish occupied a mean monthly depth of 76.3–81.6 m at temperatures as low as 4.9˚C throughout the winter before returning to shallower areas in the spring. The majority of ultrasonic detections occurred in the Bay of Fundy, but fish were detected as far as Riviere Saint-Jean, Quebec, approximately 1500 km from the Bay of Fundy (representing long-distance migratory rates of up to 44 km/day). All PSATs were first detected in the Bay of Fundy. Tags that released in February and April were found 5–21 km offshore of the Saint John Harbour, while tags that released in June were first detected in near shore areas throughout the Bay of Fundy. The substrate at winter tag release locations (estimated from backward numerical particle-tracking experiments) consisted primarily of moraines and postglacial mud substrate with low backscatter strength, indicative of soft or smooth seabed. Based on the proximity of winter tag release locations, the consistent depths observed between fish, and previous research, it is suspected that a winter aggregation exists in the Bay of Fundy. This study expands the understanding of the marine distribution and range of Atlantic sturgeon on the east coast of Canada.

## Introduction

Atlantic sturgeon, *Acipenser oxyrinchus oxyrinchus* Mitchill, 1815 are a large, migratory anadromous fish that are distributed from Florida, United States of America (USA), to Labrador, Canada [1, 2]. Atlantic sturgeon once supported a large commercial fishery [1, 3], but stocks along the east coast of North America collapsed in the late 1800’s and again in the late 1900’s due to overharvesting and habitat degradation [4–6]. The population of Atlantic sturgeon in Canada’s Maritimes region was designated as threatened by the Committee on the Status of Endangered Wildlife in Canada [7] and is currently under review by the Government of Canada for designation under the Species At Risk Act (SARA). Five Distinct Population Segments (DPS) of Atlantic sturgeon were identified as ecologically or genetically distinct in the USA [8], four of which have been listed as endangered (New York Bight, Chesapeake Bay, Carolina, and South Atlantic) and one as threatened (Gulf of Maine) under the Endangered Species Act [9, 10].

Adult Atlantic sturgeon are thought to have a spawning periodicity of 1–5 years and spend the majority of their adult lives in the marine environment [4, 11]. While in the marine environment Atlantic sturgeon form mixed-stock aggregations along the Mid-Atlantic Bight [12–15] and at a known foraging area in the summer in the Minas Basin, in the Bay of Fundy, Canada [16]. The summer aggregation in the Minas Basin consists of 6,000–14,000 Atlantic sturgeon sub-adults and adults [17]. This aggregation is comprised primarily of Atlantic sturgeon from the nearby Saint John River (~60%), but with substantial contribution from the Kennebec River, Maine (34–36%), a small proportion from the Hudson River, New York (1–2%), and less than 1% from southern US populations [18]. A pop-up satellite archival tag (PSAT) that was attached to an Atlantic sturgeon in the Hudson River in July 2007 released in the Minas Basin in June 2008 [19], indicating that Atlantic sturgeon engage in large coastal movements.

Information about over-winter behaviour and aggregation areas of Atlantic sturgeon is limited, particularly in the northern range, but trawl survey capture data suggests that Atlantic sturgeon aggregate to some degree [13, 20]. Aggregation areas of Atlantic sturgeon have been described along the Mid-Atlantic Bight at nearshore shallow areas (<40m), with predominantly sandy substrate using fisheries-dependent [12, 13, 21, 20] and fisheries-independent methods [19]. Similar shallow, sandy, and geographically concentrated winter aggregation areas have also been observed in Gulf sturgeon (*A*. *o*. *desotoi*), a subspecies of Atlantic sturgeon [22, 23]. It is likely that sand and mud substrate is commonly occupied due to the abundance of prey available [20, 24].Research has suggested that sturgeon are often closely associated with coastal features formed by bay mouths and inlets [20], and other researchers have speculated that river mouths and inlets provide concentration mechanisms for the distribution of sturgeon [25, 26].

Although winter aggregation areas are reported in shallow areas, Atlantic sturgeon have been captured or detected in deep areas offshore (>90 m; [19, 20, 27, 28]). Recently, PSATs attached to Atlantic sturgeon from the mixed stock aggregation in the Minas Basin of the Bay of Fundy, indicated that Atlantic sturgeon occupied areas with depths of 50–100 m throughout the winter (Beardsall et al. unpublished data).

Although Atlantic sturgeon occupy depths greater than 90 m, rapid vertical ascents that may result in breaching the water surface and jumping behavior have been observed. Jumping behaviour has been documented for Gulf sturgeon [23] and rapid vertical ascents were also detected in satellite archival data of Atlantic sturgeon while in the ocean (Beardsall et al. unpublished data).

We used ultrasonic telemetry and pop-up satellite archival tags (PSATs), two fisheries-independent techniques, to collect information about the marine distribution, extent of migration, and habitat occupancy of Atlantic sturgeon in their northern range following a summer migration in the Saint John River. PSATs were timed to release in the summer to provide information about yearlong activity and throughout the winter to determine potential over-winter aggregation areas. We used seasonal tag release to determine locations because it can provide more accurate fish location estimates than is possible with commonly used light-level geolocation techniques. Positional estimates at the time of tag release were further improved by implementing a novel backward numerical particle-tracking model to estimate fish position at the time of tag release. Using the estimated release locations of tags and high-resolution habitat data we provide evidence suggesting a winter aggregation area of adult Atlantic sturgeon in the Bay of Fundy. Information about the habitat and location of potential mixed-stock aggregation areas of Atlantic sturgeon is critical and must be included in trans-boundary management and regulation of the species [29].

## Materials and Methods

### Field methods

#### Capture methods and tagging

Adult Atlantic sturgeon were captured in summer 2010–2012 using gill nets 100 m x 3–4 m with 30 cm stretch mesh in the lower Saint John River, New Brunswick, Canada. All fishing was conducted according to conditions listed under license 330697 issued by Fisheries and Oceans Canada. Soak times for gill nets were 6 h in 2010, and 3–4 h in 2011 and 2012. Reduction in fishing time was conducted to limit the negative impact of capture stress on the fish. Fifty-four fish were selected based on sex, condition, and size to be tagged with Vemco V16-6X coded ultrasonic tags (length = 113.6 mm, diameter = 18.1 mm, weight in water = 19 g), V16TP-6X temperature and pressure tags (length = 117 mm, diameter = 18.1 mm, weight in water = 20.5 g), V16P-6X pressure tags (length = 117 mm, diameter = 18.1 mm, weight in water = 20 g), and Wildlife Computers MK10 (length = 175 mm, weight = 68 g) and MiniPAT (length = 115 mm, weight = 60 g) pop-up satellite archival tags. The ultrasonic tags emit bursts at a frequency of 69 kHz at 30–90 second intervals and have an estimated operational life of 1633 days.

From July 19–24 2010, 15 coded ultrasonic tags (V16-6X) and 5 coded ultrasonic tags with depth and temperature sensors (V16TP-6X) were deployed. All ultrasonic depth sensors recorded depths to 68 m (accuracy ± 0.5 m; resolution 0.3 m) and had a temperature range of -5 to 35°C (accuracy ± 0.5°C; resolution 0.15°C). In 2011, a total of 18 coded ultrasonic tags were deployed; 12 tags from June 23–29, 4 tags from July 9–10, and 2 tags on August 9. Six of these 18 ultrasonic tags had a pressure sensor to record depths. Seven Wildlife Computers MK10 pop-up satellite archival tags were deployed from August 6–9 2011. Two fish were tagged with both coded ultrasonic tags and MK10 PSATs. The MK10 data were summarized to bin temperature and depth data at 6-hour intervals from 00:00–06:00, 06:00–12:00, 12:00–18:00, and 18:00–24:00 (all times are in coordinated universal time, UTC), and were programmed to release 300 days after attachment. Data were reported as the proportion of time spent within each bin during the 6 h time periods. Upper bin limits for depths were set to 5, 10, 15, 20, 30, 40, 50, 60, 70, 80, 100, 150, 200, and >200 m. Upper bin limits for temperature were set to 2, 4, 6, 8, 10, 12, 14, 16, 18, 20, 22, 24, 26, and >26°C. Four ultrasonic tags equipped with depth sensors were deployed from July 11–19 2012 and an additional two transmitters were deployed at the same time as seven Wildlife Computers MiniPAT pop-up satellite archival tags from August 11–17 2012. MiniPATs were programmed with the upper depth bins of 5, 10, 15, 20, 30, 40, 50, 75, 100, 150, 200, >200 m, and upper temperature bins of 0, 2, 4, 6, 8, 10, 12, 16, 20, 22, 24, >24°C. Data were reported as the proportion of time spent in each bin limit using the same 6-hour intervals as the MK10 tags released in 2011. Four MiniPATs were programmed to release from February 11–20 2013, and 3 to release from April 6–12 2013. MiniPATs were programmed to release at 3-day intervals to facilitate collection and throughout the winter to provide more accurate estimates of potential over-winter locations. The PSATs also archive depth (m), temperature (°C), and light level (W/cm^2^) at 10-second (MK10) and 15-second (MiniPAT) time intervals that can be accessed if the tag is recovered following release. Collection of PSATs was conducted at sea or immediately following a tag being washed ashore. Radio and satellite transmissions from the tag occur for 3–7 days after release. After obtaining GPS coordinates from satellite transmissions, a directional radio antenna was used to detect the direction and proximity of the tag location once within range. Tags can often be found after extensive searching in the area with continued directional detections with the radio antenna.

#### Surgical procedures and sampling

Vemco ultrasonic transmitters were surgically implanted into the abdominal cavity. A 4 cm incision was made on the ventral surface on either side of the linea alba, anterior to the pelvic girdle, and a 2–0 (0.3 mm diameter) non-absorbable monofilament nylon suture with a reverse cutting needle (Ethilon 1674H) was used to attach the transmitter to the internal body wall with a single internal interrupted suture after the transmitter was inserted through the incision. The incision site was closed with 3–4 interrupted sutures. Internal sutures were used to anchor the transmitter to the internal body wall because it has been shown to reduce the potential of tag expulsion in sturgeon [30]. Pop-up satellite archival tags (PSAT) were attached using a 180 kg (tensile strength) monofilament line passed through the base of the dorsal fin, as described in [31]. We chose to conduct surgery on tagged fish without the use of anesthetics for the following reasons. 1) The high risk of human consumption following harvesting of tagged fish. Two of our tagged fish were harvested by commercial fishers within 1 week of tagging. The recapture rate of tagged fish is high: many of our tagged fish were recaptured by commercial fishers soon after they were tagged. 2) The large size of the fish in addition to the length of time required to assess the status of fish to determine necessary dosages following gill netting, the risk of overdose, and the long recovery period following anesthetizing [32, 33] precluded the use of anesthetics. 3) Our tagging procedure took less than 10 minutes and all fish were active upon release. All tagged fish were measured to the nearest cm (total length (TL), fork length (FL), and girth (G)), marked with a T-bar anchor Floy tag and implanted with a passive integrated transponder (PIT) tag (Biomark HPT12), sampled for 3 ml of blood via caudal venipuncture using a heparinized syringe (3 ml 18 gauge ½ inch Becton Dickinson & Co.), identified for sex using gonadal biopsy or by applying pressure to the abdomen causing the release of milt, and digitally imaged (Nikon Coolpix AW100 16 megapixel). All processed fish were released near the location of capture immediately after sampling procedures and tag attachment were complete. All sampling procedures used on the captured and tagged fish were conducted in accordance to guidelines and recommendations made by the Mount Allison University Animal Care and Use Committee. The sampling protocol was approved by the Mount Allison University Animal Care and Use Committee (MTA-ACC 10–16; 14–05).

### Data analysis

#### Ultrasonic data collection and filtering

**Fig 1:**
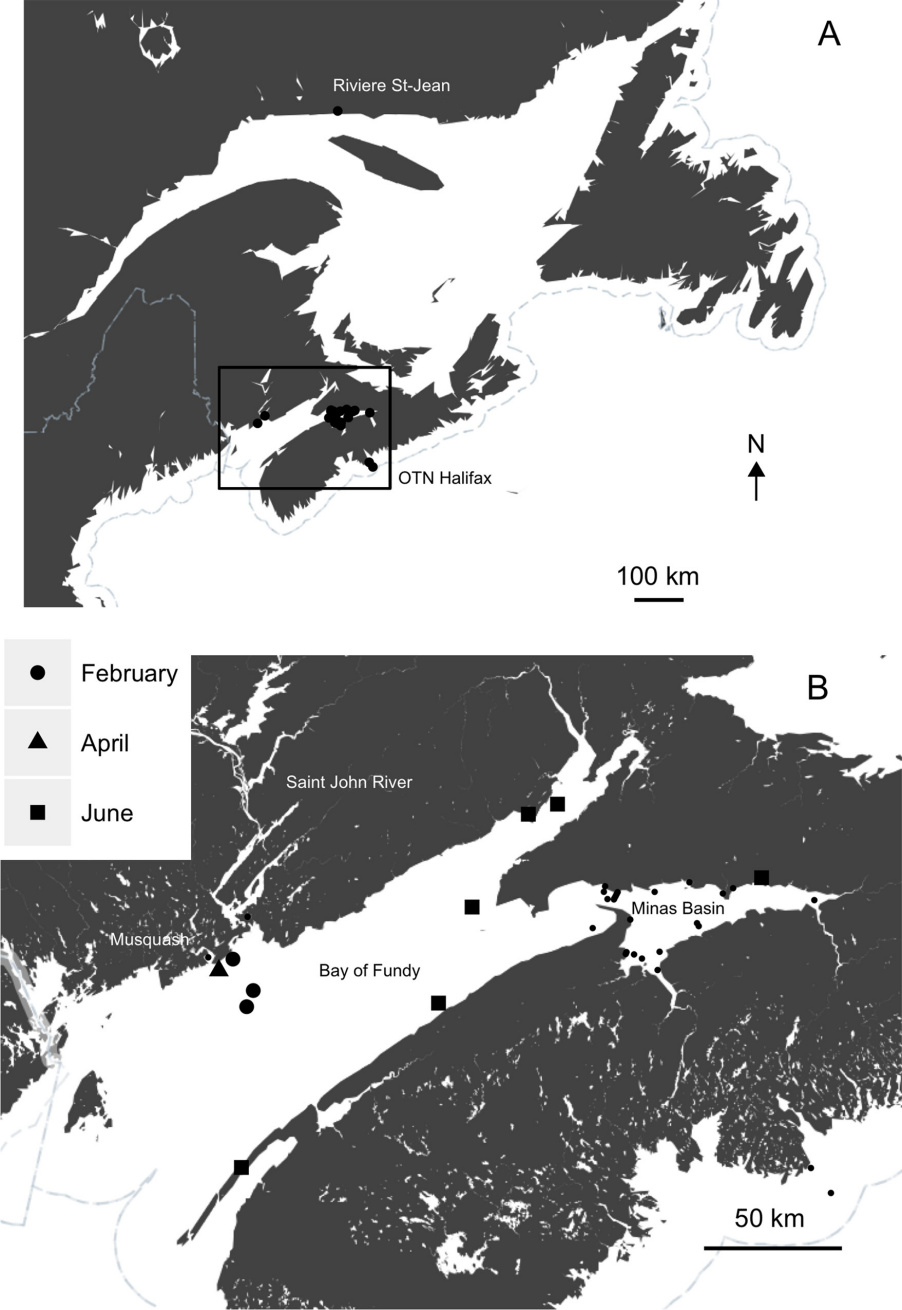
(A) Map of study region with all ultrasonic detection locations. (B) Location and time of first satellite transmission following PSAT release in the Bay of Fundy. Small black dots represent receiver locations with ultrasonic detections.

All telemetry data were inspected and filtered to remove potentially erroneous detections. Detections were removed if simultaneous detections of a transmitter occurred at geographically separated locations. If a single transmitter detection occurred at a receiver location, it was considered a false detection and omitted from the analysis. Data from ultrasonic receivers were collected by various researchers and obtained through the Ocean Tracking Network (OTN) database. Permanent receiver stations were located at the Reversing Falls, at the drainage of the Saint John River to the Bay of Fundy, the Minas Passage array, at the entrance to the Minas Basin in the upper Bay of Fundy, and the OTN Halifax array, spanning 185 km offshore near Halifax, Nova Scotia (Fig 1). Ultrasonic tags were considered inactive and removed from the analysis if they ceased movements prior to exit from the Saint John River or if detections stopped occurring and the transmitter did not pass lower receiver stations to indicate the fish left the Saint John River. It is likely that fish associated with the latter were harvested and removed from the system.

#### Seasonal distribution and habitat

The timing of exit from the Saint John River for sturgeon tagged with PSATs was determined by checking the data for abrupt decreases to temperature and depth. All fish tagged with both ultrasonic and satellite tags were used as blind tests to determine the accuracy of predicting exit time based on PSAT depth and temperature data. The timing of exit for ultrasonically tagged fish was based on the last detection at the receiver stationed at the Reversing Falls (66.08°W, 45.27°N) where the Saint John River drains to the Bay of Fundy. Mean marine depth and temperature occupancy was determined by month for all satellite tagged sturgeon. For transmitted PSAT data, the mean temperature and depth were calculated based on the proportion of time spent at each bin limit. The midpoint of each bin was calculated, multiplied by the proportion of time a fish spent at that limit for the 6 h bin period. This was then added together over all bin limits to provide the mean depth and temperature for each 6 h period. Daily means of depth and temperature were then calculated as the average of the 6 h means. This method of calculating the mean depth and temperature based on binned PSAT data has been used previously [31]. For all PSATs recovered containing archived data, full deployment plots of depth and temperature were produced to determine vertical movements and small-scale seasonal variability. Weekly variance in depth was calculated as an indicator of activity based on vertical migration. Light level data were not processed because the readings were too low to provide useful information about geolocation or hours of daylight.

#### Rapid vertical ascents

Rapid vertical ascents, potentially resulting in breaching, were frequent in PSAT archival data. A rapid vertical ascent was considered to have occurred if fast upward movement from depths >40 m to depths <10 m occurred. Depths of <10 m were used because the data are archived every 10 seconds, which results in potentially shallower depths not being recorded. The number of rapid vertical ascents was determined from November–March for all fish with archived data and the mean number of rapid vertical ascents per month was calculated for each fish.

### Backward-in-time particle drift model

A backward-in-time numerical particle-tracking model was used to estimate the longitude and latitude of a fish at the time of PSAT release for all PSATs that released during the winter. Details of the experimental design, ocean circulation model, and numerical particle tracking are given in S1 Text and S1 Table.

### Over-winter habitat classification

Over-winter habitat was classified using the 95% confidence ellipse of predicted fish locations based on the numerical particle-tracking model. The range of available depths was determined for each fish in ArcGIS using topographic charts (10 m resolution) provided by the Canadian Hydrographic Service (CHS). The available depths were compared to the depth occupied by fish at the time of PSAT release. Similarly, the proportion of each different substrate type classified by the Natural Resources Canada (NRC) was determined for the area within the confidence ellipse of each estimated tag location. The mean, minimum and maximum backscatter strength (dB) was also calculated for each predicted confidence area as a measure of the reflectivity of the sea floor. Backscatter strength data were provided at 10 m resolution from NRC. A low dB value represents relatively soft and/or smooth seabed such as muddy sand and mud, and a high dB value represents hard and/or rough seabed such as rock and gravel [34]. Low backscatter strength reaches approximately 200 dB in the Bay of Fundy, and the strongest backscatter values are up to 0 dB, providing a rudimentary distribution of bottom sediment type [34]. Tidal current and sediment mobility index (SMI) were estimated by visually approximating the range at each estimated fish location based on maps presented in [35].

## Results

### Tagging success

Fifty-four adult Atlantic sturgeon (138–213 cm FL) were tagged with ultrasonic and/or pop-up satellite archival tags. Classification of sex based on the presence of eggs or milt at capture indicated that 15 tagged individuals were female, 14 were male, and 25 were unknown. Five of 44 ultrasonic transmitters ceased movements or detections stopped occurring within the Saint John River and are not considered in this analysis. Of the 14 PSATs attached to Atlantic sturgeon (Table 1), 11 reported to the Argos satellite system. Six of 7 PSATs deployed in 2011 transmitted to the Argos satellite network at the programmed release time in 2012, and 1 was physically recovered with archived data. In 2013, 4 of 7 tags deployed in 2012 transmitted to the Argos satellite network at the programmed release time, 3 of which were physically recovered with archived data. The PSAT attached to fish 126 prematurely released and began transmitting on December 10, 2012, was recovered and archived data were downloaded. Actual release time was determined to be November 14, 2012 based on archived depth records. Of the remaining 3 tags that did not report to the Argos satellite network, the PSAT attached to fish 121 malfunctioned and was recovered on shore of the Bay of Fundy without archived data stored. Fish 124 was captured in a weir in the Minas Basin on July 15, 2014, with the PSAT still attached to the adult Atlantic sturgeon. The fish was released without the tag being removed. The whereabouts of fish 112 and the associated PSAT remain unknown.

**Table 1:** Information about Atlantic sturgeon tagged with pop-up satellite archival tags in the Saint John River in 2011 and 2012.

Fish no.	Ultrasonic tag	Date tagged	Exit from river	Scheduled release	Actual release	FL (cm)
111		07/08/2011	02/09/2011	02/06/2012	02/06/2012	165
112		06/08/2011		01/06/2012		178
113		06/08/2011	23/09/2011	02/06/2012	02/06/2012	183
114		07/08/2011	03/09/2011	03/06/2012	03/06/2012	170
115	41607	09/08/2011	16/09/2011	04/06/2012	04/06/2012	180
116		06/08/2011	05/09/2011	01/06/2012	01/06/2012	183
117	41614	09/08/2011	05/10/2011	04/06/2012	05/06/2012*	168
121		11/08/2012		14/02/2013	**	162
122		14/08/2012	15/09/2012	20/02/2013	21/02/2013	170
123	33944	11/08/2012	17/09/2012	11/02/2013	12/02/2013*	176
124		17/08/2012		12/04/2013		172
125		15/08/2012	02/09/2012	09/04/2013	10/04/2013*	181
126	33946	11/08/2012	24/09/2012	06/04/2013	14/11/2012*	168
127		14/08/2012	14/09/2012	17/02/2013	18/02/2013*	152

* Recovered with archived data

** Recovered (no satellite transmissions or archival data)

### Seasonal distribution

Ultrasonically tagged Atlantic sturgeon migrated from the Saint John River primarily between late August and mid-October (S2 Table). Table 1 indicates the timing of exit from the river for all fish tagged with PSATs based on rapid changes in depth and temperature, or ultrasonic detections when applicable. A blind comparison between ultrasonic detections at the Reversing Falls and the estimated timing of exit based on changes to depth and temperature indicated that fish 126 was predicted to enter the marine environment on the same day as detected at the Reversing Falls, while fish 115, 117, and 123 were all predicted to exit the river one day following ultrasonic detection. Based on the accuracy of the predicted time of exit from the river, analysis of coastal habitat occupancy begins at the time of predicted exit based on PSAT data, or immediately following the last ultrasonic detection at the Reversing Falls.

**Fig 2:**
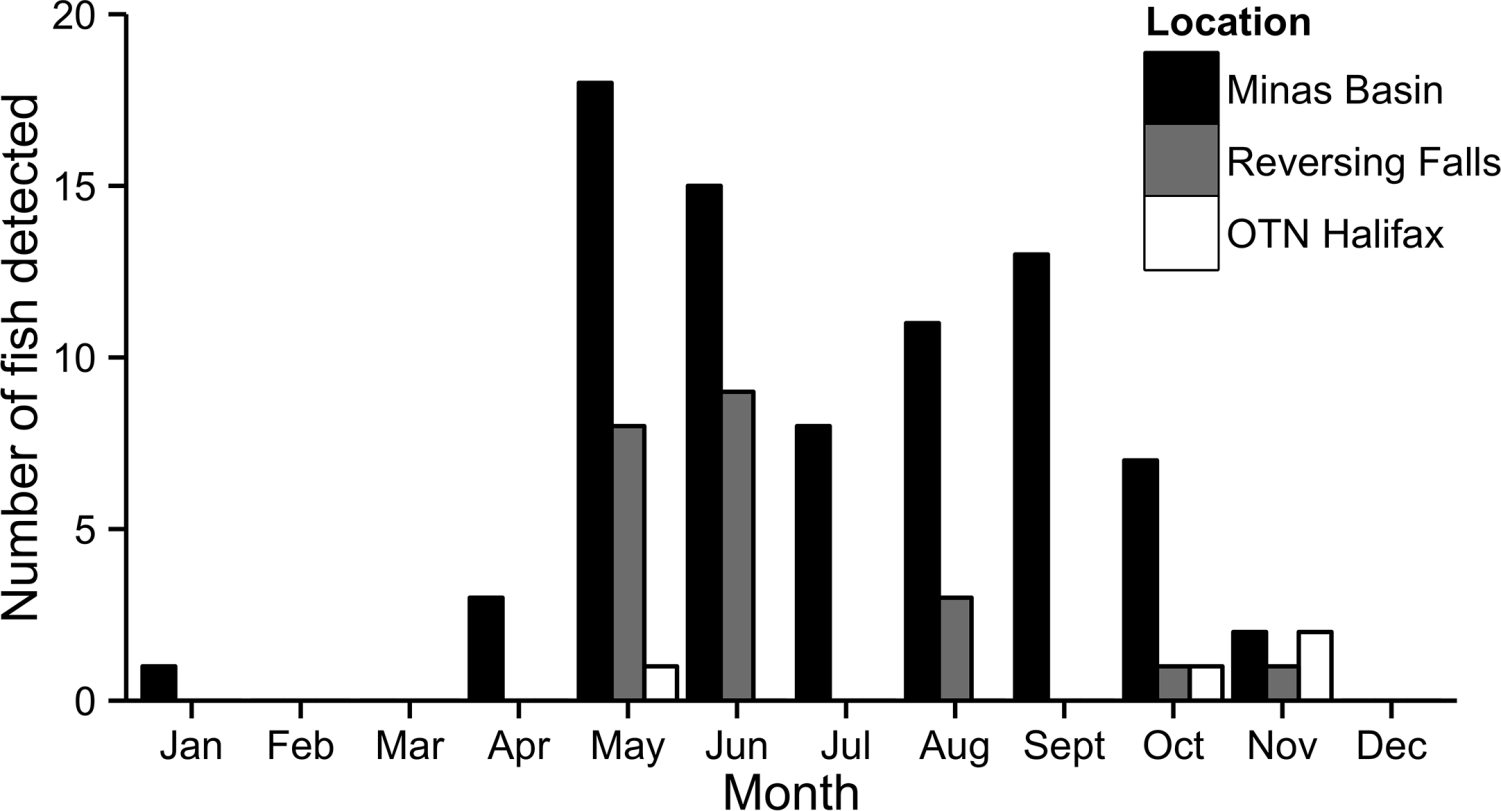
Number of tagged individuals with ultrasonic detections at permanent receiver stations in the marine environment throughout the duration of the study.

Throughout the duration of the study, 79% (31/39) of active ultrasonically tagged fish were detected in the marine environment following initial exit from the Saint John River (Table 2). Of the 31 fish detected in the marine environment, 27, or 69%, assuming no mortality in active tagged fish (39), were detected in the Minas Passage or Minas Basin. Fifty-eight percent of fish were detected in the Minas Basin the year following departure from the Saint John River, with the number of Minas Basin returns decreasing in subsequent years (Table 2). While the majority of detections occurred in the Minas Passage/Basin, four individuals were detected outside the Bay of Fundy, three on the OTN Halifax array, and one was detected in Riviere Saint-Jean, near the drainage of the Saint Lawrence River (Fig 1). The specimen with the transmitter detected in Riviere Saint-Jean travelled a minimum distance of 1500 km from Riviere Saint-Jean to the Minas Basin in ~34 days, which equates to approximately 44 km/day. Transmitters were also detected at the mouth of the Musquash Harbour and at the Reversing Falls (Fig 1), often just prior to entering the Saint John River. Ultrasonic detections at permanent receiver locations show that almost all detections occurred from spring to late fall, with only one transmitter being detected in the Minas Passage from December to March (Fig 2). All PSATs first transmitted to satellite in the Bay of Fundy (Fig 1). PSATs that released in late spring (June) first transmitted throughout inshore areas of the Bay of Fundy at known or potential feeding areas, while all PSATs that released throughout the winter months (Feb-Apr) were first detected within 20 km of each other in a small area 5–21 km offshore of the Saint John Harbour (Fig 1).

**Table 2:** Number of individuals detected at permanent ultrasonic receiver stations following exit from the Saint John River.

Year tagged (n = tagged (active))	Location	2010	2011	2012	2013
2010 (n = 20 (18))	Minas Basin	1	11	8	8
2010 (n = 20 (18))	Halifax	0	0	1	0
2010 (n = 20 (18))	Reversing Falls	0	1	0	2
2011 (n = 18 (17))	Minas Basin		1	9	5
2011 (n = 18 (17))	Halifax		2	0	0
2011 (n = 18 (17))	Reversing Falls		0	0	1
2012 (n = 6 (6))	Minas Basin			0	4
2012 (n = 6 (6))	Halifax			0	0
2012 (n = 6 (6))	Reversing Falls			0	0

### Depth occupancy and behaviour

**Fig 3:**
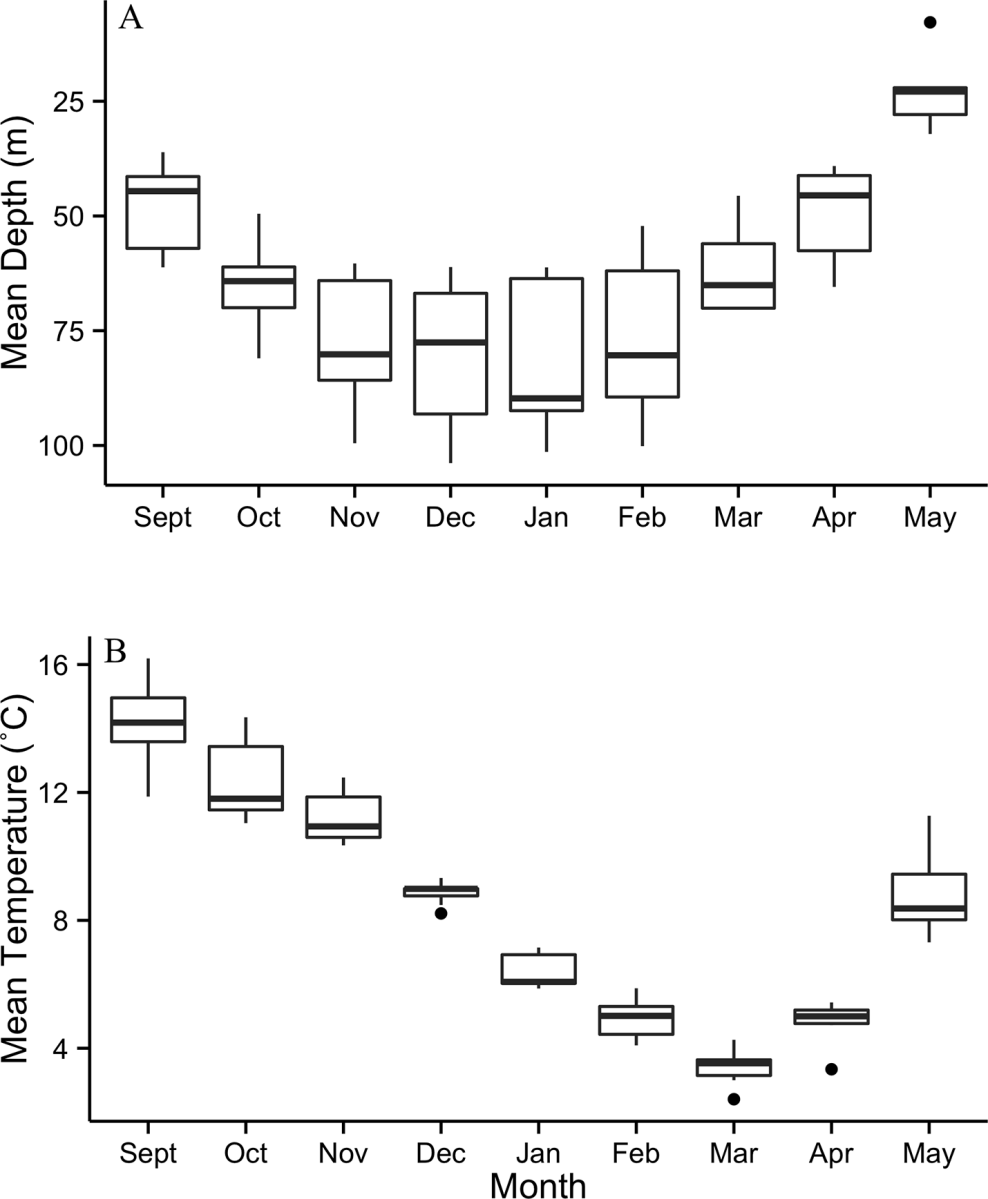
Mean (A) depth and (B) temperature by month for 10 of 11 PSATs. Data was only considered in the analysis when fish were in the marine environment.

**Fig 4:**
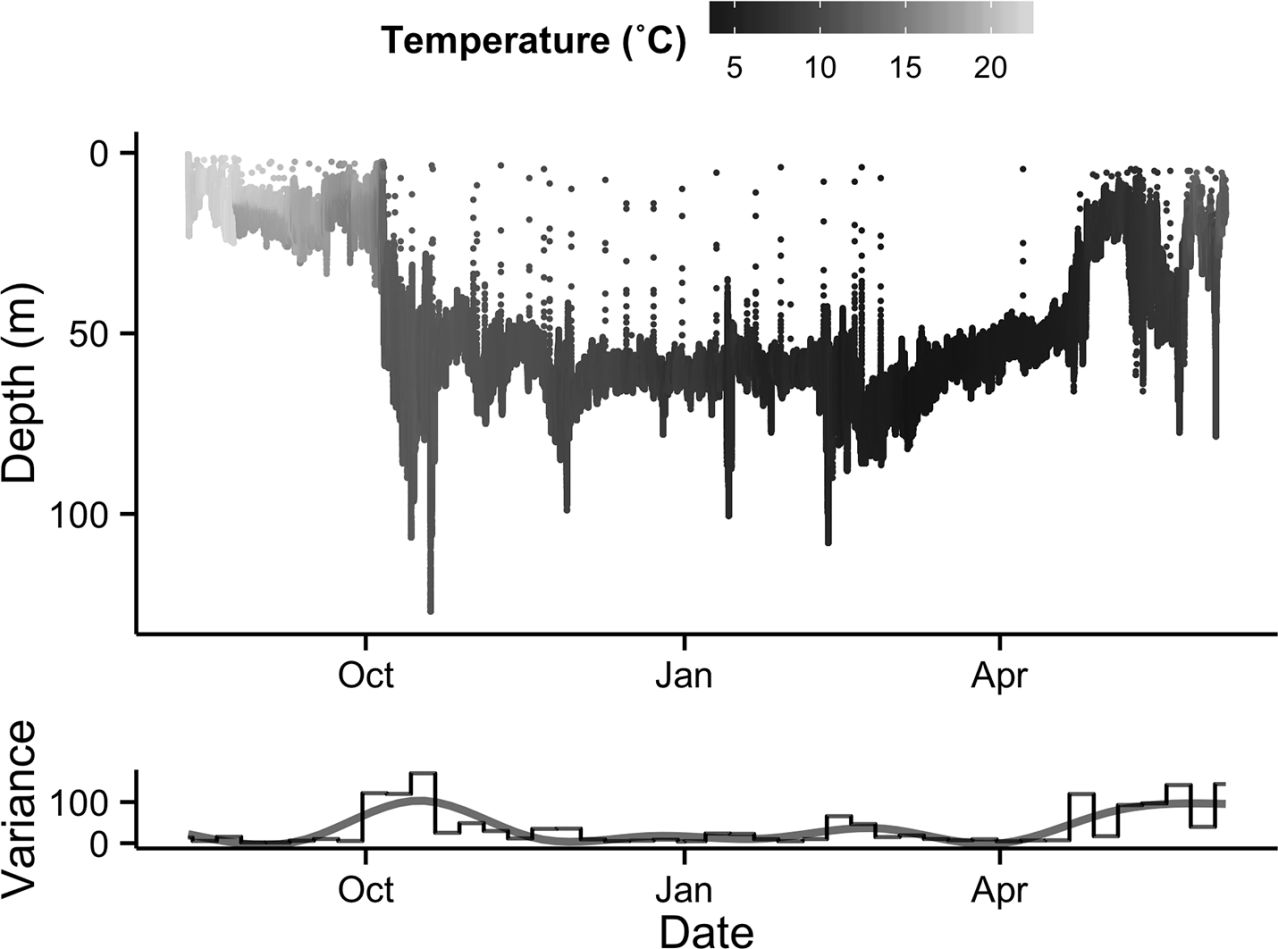
The depth and temperature profile of a tagged Atlantic sturgeon (fish 117) and weekly variance in depth for the duration of a PSAT deployment.

**Fig 5:**
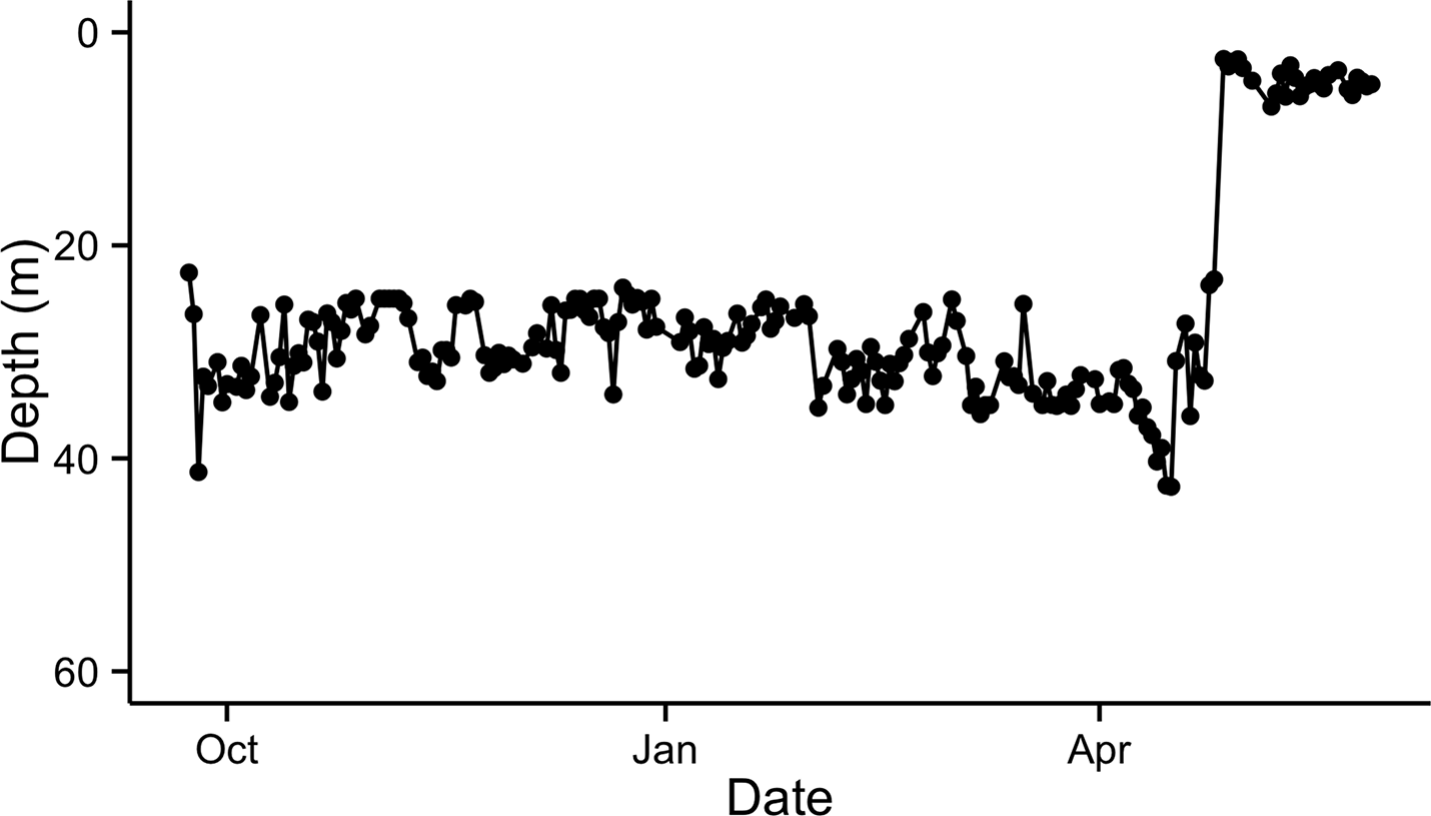
Daily mean depth of fish 113 for the duration of its PSAT deployment.

**Fig 6:**
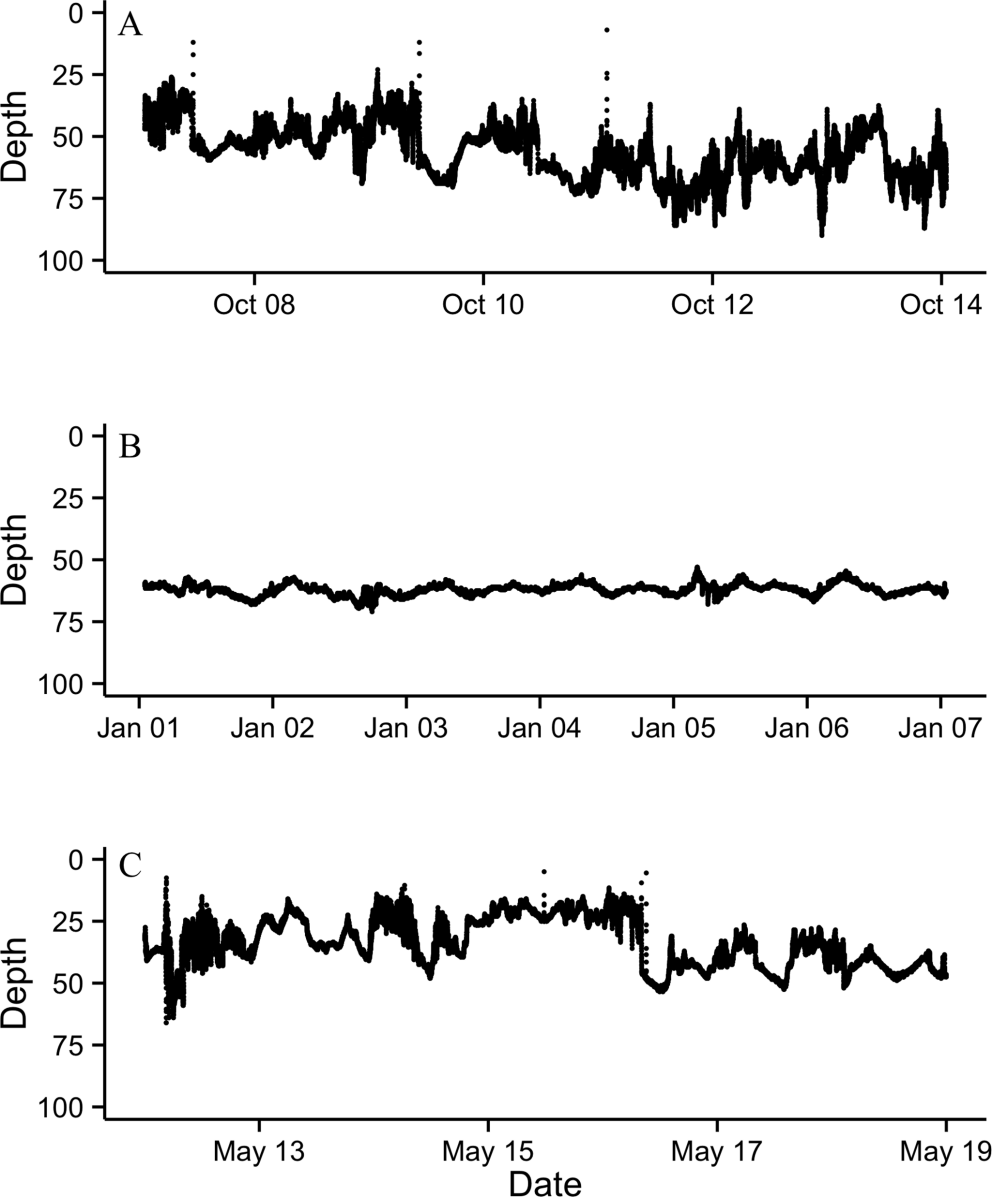
One week samples of archived data from fish 117 in (A) fall (October 7–14), (B) winter (January 1–7), and (C) spring (May 12–19).

After entering the marine environment from the Saint John River, the majority of tagged fish began a descent to depths of 64–103 m, where they remained at a fairly constant depth throughout the winter months (Fig 3). Unlike the majority of tagged fish that entered deep water immediately after exiting the river, 2 ultrasonically tagged fish were detected in the Minas Basin for 8 and 44 days following exit from the Saint John River, and fish 125 was found briefly occupying deep areas after exiting the river, returning to shallow, warmer waters before descending to a mean winter depth at 91 m (Fig 4), similar to other tagged individuals. The mean depth for 10 of 11 tagged fish ranged between 76.3 and 81.6 m for the period from November to February, and the mean temperature decreased from 11.2 to 4.9˚C during this period (Fig 3). Fish 113 did not exhibit similar depth occupancy as the other tagged individuals, remaining in shallower areas at a monthly mean depth of 26.6–32.9 m (Fig 5). The temperatures that fish 113 experienced were similar to the other tagged individuals. Archived data showed very similar depth and temperature occupancy patterns among all tagged fish (Fig 4). Weekly variance of depth indicates that after exiting the Saint John River, fish were active while descending to over-winter depth. Through the winter, fish remained at fairly constant depths, with occasional rapid vertical ascents. In February, slight increases in depth variability occur prior to a period of quiescence when water temperature was at its coldest; changes in depth at this time are attributed to tidal fluctuation (Fig 6). Following the period of quiescence, fish returned to shallow waters in the spring.

### Rapid vertical ascents

The maximum mean number of rapid vertical ascents for an individual throughout the winter was observed in fish 123 at 7.4 rapid vertical ascents per month. The maximum number of rapid vertical ascents observed in one month was 8, which occurred in both November and December. The minimum mean number of rapid vertical ascents per months was 2.4, which was observed in fish 117. Fish 117 and 125 both did not have any rapid vertical ascents in March, reflective of the lack of vertical migration observed through that time. These were the only two PSATs recovered with archived data that were attached to fish in March.

### Over-winter habitat

Using the backward-in-time numerical particle-tracking model, the actual release location and confidence ellipse of satellite tags were estimated and were found to be within a mean distance of less than 2 km from the location of first transmission. The mean (±2 SEM) of the confidence ellipse areas were 519,499 ± 238 m^2^ for the four PSATs releasing through the winter. Based on the confidence ellipses of estimated PSAT release locations, available depth ranged between 32.3 and 99.4 m. It is likely that the sturgeon were resting at or near the seafloor at the time of the PSATs’ release. There are several sources of uncertainty in our estimates of the PSAT release locations. They include: errors in the model-simulated currents (including those due to the relatively coarse horizontal resolution of the circulation model), errors in the position of the PSATs’ first contact with a satellite (which would affect the starting point of the backward particle-tracking experiments), movement of the PSATs due to winds, and numerical errors in the particle-tracking scheme.

Two tagged fish were located in areas exclusively containing moraines, while another was found in an area containing only postglacial mud and sandy mud substrate. The remaining fish was found in an area composed of 49.5% postglacial mud and sandy mud substrate, 29.4% dunes, 20% late Wisconsinan glaciomarine sediments, and <1% trapped gravel dune field. The area adjacent to all estimated fish locations is composed almost exclusively of postglacial mud and sandy mud substrate and moraines. The backscatter strength was low in all areas, with mean backscatter strength ranging between 155 and 168 dB at all locations, despite areas with of 0–200 dB occurring in the Bay of Fundy.

Sediment mobility in the area of occupancy is very low, ranging between 0.2 and 0.9 on the sediment mobility index (SMI). Tidal current speed is also low as it does not reach speeds >2 m/s in any locations where Atlantic sturgeon were located based on winter PSAT releases.

## Discussion

Adult Atlantic sturgeon migrate from the Saint John River into the Bay of Fundy in the late summer/early fall and primarily remain within the bay throughout the year, although rapid and extensive migrations outside of the bay of up to 1500 km at a minimum rate of travel of 44 km/day were observed. Similar long-distance migrations have been observed previously in Atlantic sturgeon [19], but equivalent rates of travel have not previously been documented. Other long-distance migration has been documented based on coastal bycatch of Atlantic sturgeon from the Gulf of Maine to Cape Hatteras, North Carolina that indicated that fish of Saint John River origin were caught at a mean distance of ~600 km from the river, while other populations were captured at a mean distance of nearly 1000 km from their river of origin [15].

While within the coastal environment, 76% of ultrasonically tagged individuals were detected in the Minas Basin (a known mixed-stock aggregation area [18]) in the fall, spring, or summer. PSAT release and ultrasonic detections also indicated that Atlantic sturgeon used the Minas Basin and other nearshore areas of the Bay of Fundy throughout the spring and summer. They occupied shallow, nearshore areas in the spring after occupying deep depths throughout the winter months, a pattern that has been observed in other studies of Atlantic sturgeon [19]. Tagged sturgeon in our study commonly occupied depths >90 m throughout the winter, deeper than previously recorded in Atlantic sturgeon, with a few exceptions [19, 20, 27, 28].

Our data strongly suggest that an over-winter aggregation of Atlantic sturgeon occurs in the Bay of Fundy, near the drainage of the Saint John River. All PSATs that released through the winter months were first detected in an area 5–25 km offshore and only 1 individual with an ultrasonic transmitter was detected from November–March throughout the duration of the study. Transmitted and archived PSAT data recorded deep, stable depth profiles throughout the winter. The data indicated that apart from occasional rapid vertical ascents, changes to depth occupancy are minimal throughout the winter. Comparison to bathymetric profiles shows that fish were likely resting on the bottom at depths of 60–110 m. Atlantic sturgeon tagged in the Minas Basin occupied similar depths throughout the winter (Beardsall et al. unpublished data). Genetic analysis was not conducted on the individuals tagged by Beardsall et al., but it is known that fish from as many as 3 U.S. Distinct Population Segments (DPS), in addition to the Maritimes designatable unit are present in the Minas Basin summer feeding aggregation where tags were attached [18]. Due to the similarities in depth occupancy between individuals tagged in the Saint John River and the Minas Basin, as well as genetic analysis indicating that mixed-stock aggregation areas are common throughout the species range [12–15, 18], it is possible that the over-winter area that we have defined may support fish of mixed stock origin.

The over-winter aggregation area discovered in our study is composed of substrate with low backscatter strength, representative of soft or smooth seabed such as muddy sand or mud [34]. The primary estimated areas of occupancy are, according to Natural Resources Canada’s classifications, located in postglacial mud or sandy mud and moraines. Infauna in these areas consists of polychaete worms and amphipods, basket stars, sponges, anemones, bivalves, brittle stars, and gastropods [35], which are all suitable prey items for Atlantic sturgeon. Moraine substrate is found at the deeper range of sturgeon depth occupancies observed and are described as arcuate ridges of glacial diamict up to 8 m high and 10 km long, separated by smooth seafloor [35]. Muddy and sandy substrate has been reported as the primary substrate at other winter aggregation areas of Atlantic sturgeon [12, 13, 20] and Gulf sturgeon [22], likely due to the abundance of prey available [20, 24]. The over-winter site we have identified in the Bay of Fundy extends from sand and muddy substrate to the edge of moraines where changes to substrate type occur. Other described Atlantic sturgeon aggregations have been concentrated in areas associated with specific coastal features formed by bay mouths and inlets [20]. Furthermore, the aggregation site is in close proximity to the drainage of the Saint John River into the Bay of Fundy, and it has been speculated that river mouths and inlets provide concentration mechanisms for the distribution of sturgeon [25, 26].

The sediment mobility index indicates that the substrate in this potential over-winter area is relatively stable with low tidal flow, indicating that habitat parameters likely remain consistent through time in this area; it may serve as an annual over-winter aggregation site. The geographic extent of the over-winter aggregation is unknown, but based on observed tag release locations and consistent habitat conditions, the over-winter area is likely to span a minimum width of 10 km, 5–21 km offshore. Further research should be conducted to determine the extent and genetic composition of the over-winter aggregation. Due to the proximity to the USA/Canada border and the frequency of mixed-stock aggregations of fish from U.S. DPS’s and the Maritimes population throughout their range [15, 18], this area should be used to strengthen transboundary cooperation between American and Canadian management and policy. Transboundary cooperation has previously been regarded as critical for effective conservation of Atlantic sturgeon [29]. The Bay of Fundy winter aggregation provides a unique platform to expand the current Atlantic sturgeon management policies implemented between Canada and the United States.

## Supporting Information

S1 Table Timing and location used for estimating fish position in the backwards numerical particle model. All times are in coordinated universal time (UTC).
(DOCX)

S2 Table Size of fish, date tagged, and date that the fish exited from the Saint John River for all Atlantic sturgeon tagged with ultrasonic transmitters in the Saint John River in 2010–2012. No date indicates the fish was not detected leaving the Saint John River.
(DOCX)

S1 Text Backward-in-time particle drift model. (DOCX)

## References

[1] Vladykov VD, Greeley JR. Order Acipenseroidei. Ministère de la Chasse et des Pêcheries; 1963.

[2] Scott WB, Scott MG. Atlantic fishes of Canada. Canadian Bulletin of Fisheries and Aquatic Science. 1988;219.

[3] Secor DH. Atlantic sturgeon fisheries and stock abundances during the late nineteenth century. Proceedings of the American Fisheries Society Symposium. 2002;89–100.

[4] Smith TI. The fishery, biology, and management of Atlantic sturgeon, *Acipenser oxyrhynchus*, in North America. Environmental Biology of Fishes. 1985;14(1):61–72.

[5] Smith TI, Clugston JP. Status and management of Atlantic sturgeon, *Acipenser oxyrinchus*, in North America. Environmental Biology of Fishes. 1997;48(1–4):335–346.

[6] Bain M, Haley N, Peterson D, Waldman J, Arend K. Harvest and habitats of Atlantic sturgeon *Acipenser oxyrinchus* mitchill, 1815 in the Hudson River estuary: Lessons for sturgeon conservation. Boletin-Instituto Espanol De Oceanografia. 2000;16(1/4):43–54.

[7] Convention on the Status of Endangered Wildlife in Canada (COSEWIC). COSEWIC assessment and status report on the Atlantic sturgeon *Acipenser oxyrinchus* in Canada. Ottawa. 2011;Xiii + 50pp.

[8] Atlantic Sturgeon Status Review Team (ASSRT). Status review of Atlantic sturgeon (*Acipenser oxyrinchus*) National Marine Fisheries Service and U.S. Fish and Wildlife Service, Gloucester and Hadley. Massachusetts; 2007.

[9] National Oceanic and Atmospheric Administration (NOAA). Endangered and threatened wildlife and plants; threatened and endangered status for distinct population segments of Atlantic sturgeon in the northeast region. Federal Register 77, 2012:5880–5912.

[10] National Oceanic and Atmospheric Administration (NOAA), 2012b: Endangered and threatened wildlife and plants; final listing for two distinct population segments of Atlantic sturgeon (*Acipenser oxyrinchus oxyrinchus*) in the southeast. Federal Register 77, 2012:5914–5982.

[11] Fox DA, Hightower JE, Parauka FM. Gulf sturgeon spawning migration and habitat in the Choctawhatchee River system, Alabama–Florida. Transactions of the American Fisheries Society. 2000;129(3):811–826.

[12] Savoy T, Pacileo D. Movements and important habitats of subadult Atlantic sturgeon in Connecticut waters. Transactions of the American Fisheries Society. 2003;132(1):1–8.

[13] Laney RW, Hightower JE, Versak BR, Mangold MF, Cole W, Winslow SE. Distribution, habitat use, and size of Atlantic sturgeon captured during cooperative winter tagging cruises, 1988–2006. Proceedings of the American Fisheries Society Symposium. 2007;56:167.

[14] Dunton K, Chapman D, Jordaan A, Feldheim K, O'Leary S, McKown K, et al Genetic mixed‐stock analysis of Atlantic sturgeon *Acipenser oxyrinchus oxyrinchus* in a heavily exploited marine habitat indicates the need for routine genetic monitoring. Journal of Fish Biology. 2012;80(1):207–217. doi: 10.1111/j.1095-8649.2011.03151.x 22220899 10.1111/j.1095-8649.2011.03151.x

[15] Wirgin I, Maceda L, Grunwald C, King TL. Population origin of Atlantic sturgeon *Acipenser ocyrinchus oxyrinchus* by-catch in U.S. Atlantic coast fisheries. Journal of Fish Biology. 2015;86:1251–1270. doi: 10.1111/jfb.12631 25727098 10.1111/jfb.12631 PMC4685478

[16] McLean M, Dadswell M, Stokesbury M. Feeding ecology of Atlantic sturgeon, *Acipenser oxyrinchus oxyrinchus* Mitchill, 1815 on the infauna of intertidal mudflats of Minas Basin, Bay of Fundy. Journal of Applied Ichthyology. 2013;29(3):503–509.

[17] DFO. Recovery Potential Assessment for Atlantic Sturgeon (Maritimes Designatable Unit). DFO Canadian Scientific Advisory Secretariat Science Advisory Report; 2013/022.

[18] Wirgin I, Maceda L, Waldman JR, Wehrell S, Dadswell M, King T. Stock origin of migratory Atlantic sturgeon in Minas Basin, inner Bay of Fundy, Canada, determined by microsatellite and mitochondrial DNA analyses. Transactions of the American Fisheries Society. 2012;141(5):1389–1398.

[19] Erickson D, Kahnle A, Millard M, Mora E, Bryja M, Higgs A, et al Use of pop‐up satellite archival tags to identify oceanic‐migratory patterns for adult Atlantic sturgeon, *Acipenser oxyrinchus oxyrinchus* Mitchell, 1815. Journal of Applied Ichthyology. 2011;27(2):356–365.

[20] Stein AB, Friedland KD, Sutherland M. Atlantic sturgeon marine distribution and habitat use along the northeastern coast of the United States. Transactions of the American Fisheries Society. 2004;133(3):527–537.

[21] Collins MR, Smith TI. Management briefs: Distributions of shortnose and Atlantic sturgeons in South Carolina. North American Journal of Fisheries Management. 1997;17(4):995–1000.

[22] Fox DA, Hightower JE, Parauka FM. Estuarine and nearshore marine habitat use by gulf sturgeon from the Choctawhatchee River system, Florida. Proceedings of the American Fisheries Society Symposium. 2002;28:111–126.

[23] Edwards RE, Parauka FM, Sulak KJ. New insights into marine migration and winter habitat of gulf sturgeon. Proceedings of the American Fisheries Society Symposium. 2007;56:183.

[24] Kynard B, Horgan M, Kieffer M, Seibel D. Habitats used by shortnose sturgeon in two Massachusetts rivers, with notes on estuarine Atlantic sturgeon: A hierarchical approach. Transactions of the American Fisheries Society. 2000;129(2):487–503.

[25] Dadswell M. Biology and population characteristics of the shortnose sturgeon, *Acipenser brevirostrum* LeSueur 1818 (osteichthyes: Acipenseridae), in the Saint John River estuary, New Brunswick, Canada. Canadian Journal of Zoology. 1979;57(11):2186–2210.

[26] Kynard B. Life history, latitudinal patterns, and status of the shortnose sturgeon, *Acipenser brevirostrum*. Environmental Biology of Fishes. 1997;48(1–4):319–334.

[27] Timoshkin V. Atlantic sturgeon (*Acipenser sturio* L.) caught at sea. Journal of Ichthyology. 1968;8(4):598.

[28] Dunton K, Jordaan A, Conover DO, McKown KA, Bonacci LA, Frisk MG. Marine distribution and habitat use of Atlantic sturgeon in New York lead to fisheries interactions and bycatch. Marine and Coastal Fisheres: Dynamics, Management, and Ecosystem Science. 2015;7:18–32.

[29] Apostle R, Dadswell MJ, Engler-Palma C, Litvak MK, McLean MF, Stokesbury MJW, et al Sustaining Atlantic sturgeon: Stitching a stronger scientific and governance net. Journal of International Wildlife Law & Policy. 2013;16(2–3):170–197.

[30] Crossman JA, Hammell KL, Litvak MK. Experimental examination of surgical procedures for implanting sonic transmitters in juvenile shortnose sturgeon and Atlantic sturgeon. North American Journal of Fisheries Management. 2013;33(3):549–556.

[31] Erickson DL, Hightower JE. Oceanic distribution and behavior of green sturgeon. Proceedings of the American Fisheries Society Symposium. 2007;56:197.

[32] Damon-Randall K, Bohl R, Bolden S, Fox D, Hager C, Hickson B, Hilton E, et al. Atlantic sturgeon research techniques. NOAA Technical Memorandum NMFS-NE-215 2010:64.

[33] Balazik MT, Langford BC, Garman GC, Fine ML, Stewart JK, Latour RJ, et al Comparison of MS-222 and electronarcosis as anesthetics on cortisol levels in juvenile Atlantic sturgeon. Transactions of the American Fisheries Society. 2013:142(6):1640–1643.

[34] Todd BJ, Shaw J, Li MZ, Kostylev VE, Wu Y. Distribution of subtidal sedimentary bedforms in a macrotidal setting: The Bay of Fundy, Atlantic Canada. Continental Shelf Research. 2014;83:64–85.

[35] Shaw J, Todd B, Li M. Seascapes, Bay of Fundy, offshore Nova Scotia/New Brunswick. Geological Survey of Canada Open File 7028; 2012.

