# Supplementary material for: Oceanic Distribution, Behaviour, and a Winter Aggregation Area of Adult Atlantic Sturgeon, Acipenser oxyrinchus oxyrinchus, in the Bay of Fundy, Canada
Source: PLoS One. 2016 Apr 4;11(4):e0152470. doi: 10.1371/journal.pone.0152470 (PMC4820111; doi:10.1371/journal.pone.0152470)
Supplement: S1 Table — All times are in coordinated universal time (UTC). (DOCX) [file pone.0152470.s001.docx]

S1 Table. Timing and location used for estimating fish position in the backswards numerical particle model. All times are in coordinated universal time (UTC).

| Fish no. | First satellite detection | | Arrival at ocean surface | Start of experiment | End of experiment |
| --- | --- | --- | --- | --- | --- |
|  | Location | Time |  |  |  |
| 123 | 66.0500^o^W 45.0336^o^N | 12/02/2013 00:25 | 12/02/2013 00:04 | 12/02/2013 00:27 | 12/02/2013 00:03 |
| 127 | 66.0800^o^W 44.9817^o^N | 18/02/2013 01:41 | 18/02/2013 00:48 | 18/02/2013 01:42 | 18/02/2013 00:48 |
| 122 | 66.1420^o^W 45.1350^o^N | 21/02/2013 01:16 | 20/02/2013 20:00 | 21/02/2013 01:18 | 20/02/2013 20:00 |
| 125 | 66.2064^o^W 49.0978^o^N | 10/04/2013 00:47 | 10/04/2013 00:20 | 10/04/2013 00:48 | 10/04/2013 00:18 |
