# Supplementary material for: Oceanic Distribution, Behaviour, and a Winter Aggregation Area of Adult Atlantic Sturgeon, Acipenser oxyrinchus oxyrinchus, in the Bay of Fundy, Canada
Source: PLoS One. 2016 Apr 4;11(4):e0152470. doi: 10.1371/journal.pone.0152470 (PMC4820111; doi:10.1371/journal.pone.0152470)
Supplement: S2 Table — No date indicates the fish was not detected leaving the Saint John River. (DOCX) [file pone.0152470.s002.docx]

S2 Table. Size of fish, date tagged, and date that the fish exited from the Saint John River for all Atlantic sturgeon tagged with ultrasonic transmitters in the Saint John River in 2010-2012. No date indicates the fish was not detected leaving the Saint John River.

| Fork Length (cm) | Date of tagging | Exit from River |
| --- | --- | --- |
| 163 | July 19/2010 | September 29/2010 |
| 155 | July 19/2010 | September 19/2010 |
| 188 | July 19/2010 | November 11/2010 |
| 174 | July 20/2010 | September 15/2010 |
| 161 | July 20/2010 | August 13/2010 |
| 168 | July 20/2010 | August 15/2010 |
| 178 | July 20/2010 |  |
| 155 | July 20/2010 | July 29/2010 |
| 189 | July 20/2010 |  |
| 161 | July 21/2010 | September 17/2010 |
| 138 | July 21/2010 | September 12/2010 |
| 187 | July 21/2010 |  |
| 178 | July 22/2010 | September 15/2010 |
| 140 | July 22/2010 |  |
| 168 | July 23/2010 | September 24/2010 |
| 157 | July 23/2010 |  |
| 146 | July 23/2010 |  |
| 157 | July 24/2010 |  |
| 145 | July 24/2010 |  |
| 198 | July 24/2010 | August 28/2010 |
| 147 | July 9/2011 | July 19/2011 |
| 145 | July 9/2011 | September 8/2011 |
| 173 | June 23/2011 | September 11/2011 |
| 178 | June 24/2011 | September 5/2011 |
| 170 | June 24/2011 | July 27/2011 |
| 163 | June 24/2011 |  |
| 183 | June 25/2011 | September 24/2011 |
| 150 | June 25/2011 | September 4/2011 |
| 155 | June 26/2011 | August 1/2011 |
| 152 | June 26/2011 | September 4/2011 |
| 165 | June 26/2011 |  |
| 183 | June 27/2011 | August 6/2011 |
| 147 | June 28/2011 | September 7/2011 |
| 150 | June 29/2011 |  |
| 160 | July 10/2011 | August 30/2011 |
| 152 | July 10/2011 | August 31/2011 |
| 180 | August 9/2011 | September 16/2011 |
| 168 | August 9/2011 | October 5/2011 |
| 143 | July 11/2012 | August 17/2012 |
| 163 | July 12/2012 | September 10/2012 |
| 149 | July 17/2012 | August 20/2012 |
| 177 | July 18/2012 | September 28/2012 |
| 176 | August 11/2012 | September 17/2012 |
| 168 | August 11/2012 | September 24/2012 |
