# Supplementary material for: Oceanic Distribution, Behaviour, and a Winter Aggregation Area of Adult Atlantic Sturgeon, Acipenser oxyrinchus oxyrinchus, in the Bay of Fundy, Canada
Source: PLoS One. 2016 Apr 4;11(4):e0152470. doi: 10.1371/journal.pone.0152470 (PMC4820111; doi:10.1371/journal.pone.0152470)
Supplement: S1 Text — (DOCX) [file pone.0152470.s003.docx]

**S1 Text. Backward-in-time particle drift model**

**Experimental design**

A backward-in-time numerical particle-tracking model was used to estimate the longitude and latitude of a fish at the time of PSAT release for all PSATs that released during the winter. This model combined 1) time-dependent, three-dimensional (3D) ocean currents simulated by a numerical circulation model and 2) a numerical particle-tracking scheme that calculated the movement of particles being passively transported by the simulated ocean currents. For this application, the particles’ movement was calculated going backward in time, in order to estimate the position of a PSAT at a point in time (i.e. time of release) given its position at a later time (i.e. time of first contact with the satellite). Time of release from the fish was recorded and uploaded to the Argos satellite system and was confirmed using depth records from the tags. (For our purposes, we assumed that a PSAT’s ascent from the fish’s location to the surface was instantaneous.) Four numerical experiments were conducted corresponding to each PSAT that released from February to April. In each experiment, particles were released horizontally 5 m apart in a rectangular array that spanned approximately 90 m in each direction and was centered on the location of first satellite detection. As a result, approximately 340 particles were released in each experiment. The particles maintained a constant depth of 0.1 m below the ocean surface throughout each experiment. The ocean current field simulated by the ocean circulation model was saved at three-minute intervals. Each experiment began at the closest three-minute increment after the time of first satellite detection, and ended at the closest three-minute increment before the time of the tag’s arrival at the ocean surface (i.e. the period of each experiment is chosen to bracket the period of interest).

**Ocean circulation model**

The numerical ocean circulation model used in this study simulates time-dependent, 3D circulation fields, of which the horizontal components were used in the numerical particle-tracking scheme. The model was based on the Princeton Ocean Model [1] and followed the design of [2]. The model had a horizontal resolution of 1/16˚ and covers the region of 71.5˚W-56˚W and 38.5˚N-52˚N, which includes the Gulf of St. Lawrence, the Scotian Shelf, the Bay of Fundy-Gulf of Maine system and adjacent deep waters. The model used the terrain-following sigma vertical coordinate system, with 40 sigma-layers. The minimum water depth in the model bathymetry was set to be 10 m and the maximum depth is 4500 m.

The model temperature and salinity were nudged toward gridded climatologies of observed temperature and salinity at the mean, annual, and semi-annual time scales using the spectral nudging method [3]. Outside of these time scales, the model’s dynamics were not directly affected by the nudging method and the model state variables evolve prognostically. In addition, the semi-prognostic method [4] was also used to reduce the seasonal drift in the simulated circulation.

At the sea surface, the model was forced by atmospheric pressure and wind stress. The atmospheric pressure was extracted from the North American Regional Reanalysis dataset (NARR; [5]) and the wind stress was calculated from the near-surface wind velocity in the NARR dataset. The circulation model was also forced at the surface by the net heat flux, calculated from the model sea surface temperature and NARR fields of air temperature, cloud cover, downward shortwave radiation flux, and precipitation.

Along lateral open boundaries of the circulation model, three types of fields were specified: (1) sea level and depth-mean currents produced by a barotropic model, (2) sea level and depth-mean currents for five tidal constituents, and (3) depth-dependent temperature, salinity, and currents simulated by a circulation model covering the northwest Atlantic [6]. The model was initialized with monthly-mean climatological fields of temperature and salinity. Rivers were represented by idealized channels cut into the model’s coastline and daily values of river discharge were specified at the heads of these channels.

In this study, the ocean circulation model was started on January 1, 2012, and the 3D current fields were archived every three minutes during four periods in 2013 when a PSAT was drifting at the ocean surface.

**Numerical particle-tracking**

Although the ocean circulation model described above produces 3D current fields, only the two horizontal components were used in this study because the numerically-tracked particles were programmed to maintain a constant depth (0.1 m) below the surface to represent movement of floating PSATs.

The numerical particle-tracking scheme was run backwards in time to estimate the position of the PSAT immediately after detaching from the fish and reaching the surface, which occurred up to 5.3 hours before the first satellite detection. Backward numerical particle-tracking can be expressed as the sum of movements due to currents (whose signs have been reversed) and a “random walk” component:

(1)

where and are the two-dimensional (2D) position vectors of a particle at times *t* and *t-Δt* respectively, is the 2D vector of ambient currents, and is a 2D random walk component that represents sub-grid scale turbulence and other unresolved local processes [7]. The horizontal advection of particles due to currents was calculated using the fourth-order Runge-Kutta method [8]. The components of , which are expressed as *δx* and *δy* in the *x* and *y* directions respectively, can be expressed as [9]:

, (2)

where and are random numbers in the range [-1,1] and *K_h_* is the horizontal eddy diffusivity coefficient. Values of *K_h_* estimated from field experiments have a large range. For example, surface drifter experiments conducted in the coastal waters of Taiwan estimated *K_h_* to be between 0.2 and 5 m^2^ s^-1^ [10]. In this study, *K_h_* is set to 10 m^2^ s^-1^ to minimize mismatches between depths recorded by the PSATs and water depths within the range of estimated positions at which the PSATs were released.

At the end of each backward particle-tracking experiment (i.e. at the time of the PSAT’s release), we calculated the radius of a circle within which 95% of the particles are located. This circle will be referred to as the 95% confidence ellipse in the following discussion.

**References**

1. Mellor GL. Users guide for a three-dimensional, primitive equation, numerical ocean model. Princeton, NJ: Princeton University. 2005;52 p.

2. Ohashi K, and Sheng J. Influence of the St. Lawrence River discharge on the circulation and hydrography in Canadian Atlantic waters. Continental Shelf Research. 2013;58: 32-49. doi:10.1016/j.csr.2013.03.005.

3. Thompson KR, Ohashi K, Sheng J, Bobanovic J, Ou J. Suppressing bias and drift of coastal circulation models through the assimilation of seasonal climatologies of temperature and salinity. Continental Shelf Research. 2007;27: 1303-1316.

4. Sheng J, Greatbatch RJ, Wright DG. Improving the utility of ocean circulation models through adjustment of the momentum balance. Journal of Geophysical Research. 2001;106:16711-16728.

5. Mesinger F, DiMego G, Kalnay E, Mitchell K, Schaffran PC. North American Regional Reanalysis. Bulletin of American Meteorological Society. 2006;87: 343-360. doi:10.1175/BAMS-87-3-343.

6. Urrego-Blanco J, Sheng J. Interannual variability of the circulation over the eastern Canadian shelf. Atmosphere Ocean. 2012;50: 277-300. do3i:10.1080/07055900.2012.680430.

7. Shan S, Sheng J, Greenan BJW. Modelling study of three-dimensional circulation and particle movement over the Sable Gully of Nova Scotia. Ocean Dynamics. 2014;64:117-142.

8. Press WH, Teukolsky SA, Vetterling WT, Flannery BP. Numerical Recipes in FORTRAN (2nd ed.). Cambridge: Cambridge University Press. 1992;xxvi+963p.

9. Taylor GI. Diffusion by continuous movements. Proceedings of the London Mathematical Society. 1922;s2-20: 196-212.

10. Tseng RS. On the dispersion and diffusion near estuaries and around islands. Estuaries, Coastal and Shelf Science. 2002;54: 89-100.
